# Loss of Sphingosine Kinase Alters Life History Traits and Locomotor Function in *Caenorhabditis elegans*

**DOI:** 10.3389/fgene.2017.00132

**Published:** 2017-09-21

**Authors:** Jason P. Chan, Jaylene Brown, Brandon Hark, Abby Nolan, Dustin Servello, Hannah Hrobuchak, Trisha A. Staab

**Affiliations:** Department of Biology, Juniata College Huntingdon, PA, United States

**Keywords:** sphingosine kinase, sphingolipids, health span, *C. elegans*, life history traits, aging, stress, neuromuscular function

## Abstract

Sphingolipid metabolism is important to balance the abundance of bioactive lipid molecules involved in cell signaling, neuronal function, and survival. Specifically, the sphingolipid sphingosine mediates cell death signaling, whereas its phosphorylated form, sphingosine-1-phosphate (S1P), mediates cell survival signaling. The enzyme sphingosine kinase produces S1P, and the activity of sphingosine kinase impacts the ability of cells to survive under stress and challenges. To examine the influence of sphingolipid metabolism, particularly enzymes regulating sphingosine and S1P, in mediating aging, neuronal function and stress response, we examined life history traits, locomotor capacities and heat stress responses of young and old animals using the model organism *Caenorhabditis elegans*. We found that *C. elegans sphk-1* mutants, which lack sphingosine kinase, had shorter lifespans, reduced brood sizes, and smaller body sizes compared to wild type animals. By analyzing a panel of young and old animals with genetic mutations in the sphingolipid signaling pathway, we showed that aged *sphk-1* mutants exhibited a greater decline in neuromuscular function and locomotor behavior. In addition, aged animals lacking *sphk-1* were more susceptible to death induced by acute and prolonged heat exposure. On the other hand, older animals with loss of function mutations in ceramide synthase (*hyl-1*), which converts sphingosine to ceramide, showed improved neuromuscular function and stress response with age. This phenotype was dependent on *sphk-1*. Together, our data show that loss of sphingosine kinase contributes to poor animal health span, suggesting that sphingolipid signaling may be important for healthy neuronal function and animal stress response during aging.

## Introduction

Sphingolipids comprise up to 20% of membrane lipids and include sphingomyelin (SM), ceramide (CER), sphingosine (SPH), and sphingosine-1-phosphate (S1P) (Alvarez et al., 2007; Hannun and Obeid, 2008). Sphingolipids function both in membrane support roles and as bioactive signaling molecules that mediate myriad cell activities. The balance between the cellular levels of S1P and CER/SPH is referred to as the sphingolipid rheostat. Cellular S1P promotes cell growth, proliferation, and survival, whereas elevated SPH and CER mediate apoptosis (Spiegel and Milstien, 2003; Hannun and Obeid, 2008). Thus, the genes coding the metabolic enzymes that regulate the balance of these lipids are important for cellular function, stress response, development and survival (Hait et al., 2006; Van Brocklyn and Williams, 2012; Romero-Guevara et al., 2015).

The gene sphingosine kinase, which codes for the enzyme that phosphorylates SPH and alters SPH/CER and S1P balance, can affect age-related decline in normal neuronal function and longevity. For example, sphingolipids regulate chronological lifespan in yeast, worms, and mammals (Huang et al., 2014), and lower tissue levels of S1P are a risk factor for many age-related disorders, including Alzheimer's disease, coronary artery disease, immune dysfunction, diabetes, and obesity (Spiegel and Milstien, 2003; He et al., 2010; Maceyka et al., 2012; Kawabori et al., 2013; Levkau, 2013). Conversely, ceramide accumulates in aged animals, including worms and humans and may lead to shorter lifespans (Cutler et al., 2014; Huang et al., 2014). For example, elevated ceramide in *C. elegans* reduces lifespan, whereas mutants lacking acid sphingomyelinase, an enzyme that breaks down sphingomyelin to ceramide, have lengthened lifespan (Kim and Sun, 2012; Cutler et al., 2014). However, it is unclear how altered sphingosine kinase function impacts lifespan or health span. Lifespan is the duration of an animal's life whereas health span is the duration of years of healthy living in animals. The examination of genes affecting sphingolipid metabolism will be important to identifying signaling pathways promoting healthy aging.

The ability to maintain movement and stress response is vital to organismal survival and longevity, and the activity of sphingosine kinase may be important for this. Reducing sphingosine kinase activity and S1P levels decrease stress resilience and mobility in animals (Chung et al., 2000; Hannun and Obeid, 2008; Maceyka et al., 2012; Van Brocklyn and Williams, 2012). Sphingosine kinase is known to facilitate neurotransmitter release at neuromuscular junctions and central brain tissues, suggesting that sphingosine kinase may play a role in aged animals to dampen decline in cognitive or motor function (Brailoiu et al., 2002; Okada et al., 2009; Chan et al., 2012; Shen et al., 2014). Indeed, activating S1P signaling with the modulator fingolimod promotes motor function and reduces brain atrophy in mouse models (Di Pardo et al., 2014). Further studies investigating sphingosine kinase in aged neurons are warranted to better understand healthy neuronal aging.

Lipid metabolism is known to alter life history traits such as development, reproduction, and lifespan (Branicky et al., 2010), but the pathways regulating qualitative measures of healthy aging are less understood. Here, we aim to examine the enzymes that regulate the sphingolipid rheostat and their role in mediating healthy aging. To accomplish this, we examined life history traits, including development, reproduction, and lifespan, and maintenance of motor performance using *Caenorhabditis elegans* with genetic alterations in sphingolipid metabolic enzymes. Our study shows that *C. elegans* mutants lacking sphingosine kinase have reduced lifespan, shorter body sizes, and smaller brood sizes. Furthermore, mutants have a greater age-related decline in neuromuscular function and motor performance. Thus, regulating enzymes mediating sphingolipid metabolism may be important for healthy aging, and loss of sphingosine kinase may exacerbate age related neurological dysfunction.

## Materials and methods

### *C. elegans* strains

All strains were outcrossed at least 4x, grown on nematode growth media (NGM), and cultured using standard methods at room temperature. Worms were stored in a cabinet in a temperature controlled room. To confirm that temperatures did not vary greatly, we collected data at 15 min intervals using a temperature data logger (Elitech). The average temperature was 20.9°C over a 5-day period (range of 20.5°C to 21.5°C). Plates were seeded with *E. coli* HB101 because sphingolipid mutants grow better on this bacteria compared to OP50. For lifespan and aging studies, NGM plates were supplemented with 50 μM 5-Fluoro-2′-deoxyuridine (FUdR, Alfa Aesar). FUdR is used to inhibit DNA synthesis, preventing eggs from hatching. The following strains were provided by the CGC, which is funded by NIH Office of Research Infrastructure Programs (P40 OD010440): *sphk-1(ok1097), hyl-1(gk203), hyl-2(gnv1), T10B11.2(ok1252)*. The wild type strain was *N2* Bristol. Other strains used include OJ802(*vjEx315;sphk-1(ok1097)*), JPC14(*sphk-1(ok1097);hyl-1(gk203)*), and JPC15(*sphk-1(ok1097);hyl-2(gnv1)*).

### Life history traits

Lifespan was measured using animals grown on NGM/*HB101* plates at room temperature. Age-matched L4 animals were transferred to NGM plates containing 5'-fluorodeoxyuridine (FUdR; 50 μM) for all genotypes examined. At least three replicate plates, or trials, of 20–25 worms were examined for each genotype, except *sphk-1;hyl-2*. Worms were counted every other day and were transferred to fresh plates every 4–5 days, or when necessary. Animals were scored as dead if they did not respond to a gentle prodding with a platinum rod. Animals lost from crawling up the side of petri dishes and bagged animals were censored from the data set. Mean life span was also determined by calculating the last day on which a live worm was observed. All sample sizes, censored animals, and trial numbers are given in **Table 2**.

For brood size, individual L4 hermaphrodite worms were placed on NGM/*HB101* plates, and transferred every 24 h for 5 consecutive days onto fresh NGM/*HB101* plates, until they were 7 days old. Surviving progeny from each plate were counted 2 days after the transfer or removal of the parent worm. A minimum of 5 plates were used for each strain. Eggs that did not hatch were not counted, leading to a modified, surviving brood size count.

**Figure 1:**
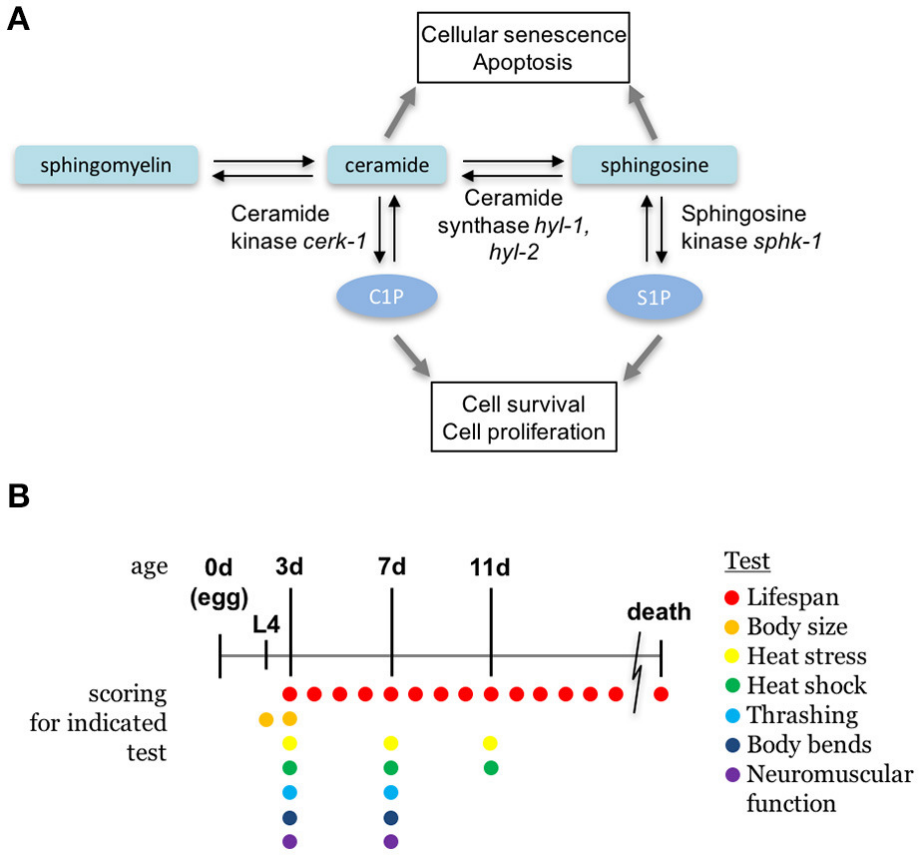
Sphingolipid metabolism pathway. **(A)** The sphingolipid signaling pathway is composed of sphingomyelin, ceramide, sphingosine, and sphingosine-1-phosphate, and are interconverted by metabolic enzymes. *C. elegans* orthologs of enzymes examined in this paper are indicated. **(B)** A diagram depicting the ages of animals during the scoring days of experiments. All ages are relative to egg lay and the presence of colored circles indicate that a respective test was performed on that day.

For body size, worms were synchronized by allowing gravid adult to lay eggs in a 2-h window. After removal of the parent, progeny were allowed to grow for 48 and 72 h (Figure 1B). Body sizes were measured using images of worms, captured using an Infinity 2 camera and Infinity Analyze software (Lumenera), and images were analyzed using ImageJ (NIH). At least 30 worms were measured for body length, with length being from the tip of the head to the end of the tail.

### Locomotion assays

For experiments analyzing locomotor capacity, worms were synchronized by allowing gravid adults to lay eggs in a 2-h window, and eggs were allowed to grow to 3 days or 7 days, depending on the experiment (Figure 1B). For experiments of neuromuscular function, aged wild type and mutant hermaphrodites were examined for their sensitivity to an acetylcholine esterase inhibitor, aldicarb (Sigma). Three plates of 20 age-matched animals per genotype were transferred onto NGM plates containing 1 mM aldicarb. Movement was scored every 15 min for 2 h, and genotypes were blind to the scorer. For analyses of 7-day-old animals, worms were transferred to FUdR-containing plates at 3 days to prevent new progeny from developing.

Thrashing and body bend assays were performed to test for locomotor capacity. On test days, animal behavior was recorded by video using an Infinity 2 camera (Lumenera). For thrashing assays, individual worms were added to M9 solution, allowed to equilibrate for 1 min, and scored the next 15 s for the number of thrashes (this number multiplied by four to get thrashes per minute). For body bending assays, individual worms were transferred onto a NGM plate containing *E. coli (HB101)*, and scored the next 30 s for the number of bends (this number was doubled to get body bends per minute).

### Stress assays

Heat stress assays were performed on adult hermaphrodites grown on NGM/*HB101*, as described elsewhere (Keith et al., 2014). Briefly, animals from synchronized egg lays, as described above, were aged to 3, 7, or 11 days after hatching (Figure 1B). For heat stress assays, age-matched worms were incubated at 35°C and scored for survival at 2, 3, 4, 6, and 9 h. For heat shock assays, age-matched adult hermaphrodites were incubated at 37°C for 90 min, and counted for survival 12 h later. For both assays, worms were considered dead when they no longer responded to gentle prodding in the head region. Aged animals tested at 7 or 11 days were transferred to FUdR plates at 3 days to prevent new progeny from developing.

### Statistical analysis

For analyses of lifespan and of survival to stress over time, a Kaplan-Meier estimator was used using the program OASIS (Yang et al., 2011). All other statistical analysis was completed using R statistical package. Statistical differences were determined using one- or two-way ANOVAs, followed by Tukey's HSD *post-hoc* tests for pairwise comparisons.

## Results

### Altering sphingolipid metabolism enzymes affects longevity, reproduction, and development

**Figure 2:**
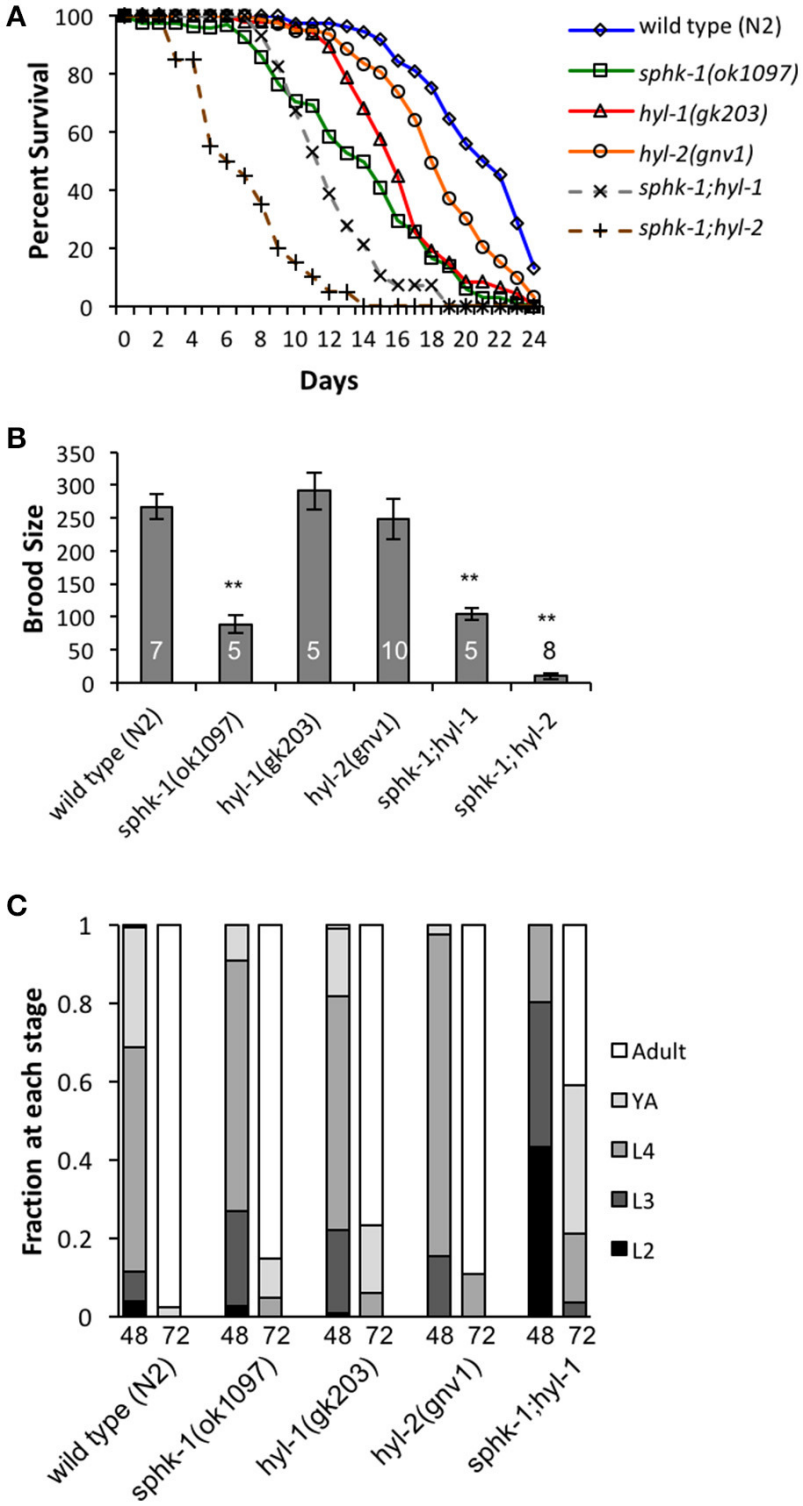
Sphingolipid enzymes regulate life history traits. **(A)** Lifespan curves of wild type, *sphk-1, hyl-1, sphk-1;hyl-1, hyl-2*, and *sphk-1;hyl-2*. For all animals, all days are counted relative to egg lay. Significant differences in lifespan and mean survival were determined using a Kaplan-Meier estimator and Log-rank tests (Yang et al., 2011). **(B)** Brood size of animals of the indicated strain (*n* values are indicated). L4 animals were singled onto individual plates every 24 h for 6 days, and hatched eggs were counted 2 days after removal of the parent, representing a surviving population. For brood size, ^**^p < 0.001 and significant differences were determined by one-way ANOVA and Tukey's HSD *post-hoc* tests. **(C)** Developmental level of wild type, *sphk-1, hyl-1, sphk-1;hyl-1*, and *hyl-2* at 48 and 72 h after a 2 h egg lay. Error bars are ± SEM.

The conserved sphingomyelin pathway (Figure 1A and Table 1) links external environmental cues with an intracellular signaling network that mediates cell responses and cell survival (Hannun and Obeid, 2008). The metabolic enzymes function to balance the cellular levels of ceramide (CER)/sphingosine (SPH) vs. S1P; this balance often determines the difference between cellular apoptosis or survival and growth. Thus, we examined whether animals lacking sphingosine kinase displayed differences in life history traits compared to wild type animals. Our experiments were completed with animals grown on *HB101 E. coli* because we previously observed that sphingosine kinase mutants appear healthier when fed *HB101* compared to *OP50* (personal observation). It is possible that the increased carbohydrate observed in *HB101*, compared to *OP50* bacterial strains, promotes a healthier state (Brooks et al., 2009). Interestingly, *C. elegans* fed *HB101* may have less stored fat, slightly increased lifespans, and increased the rate of germline proliferation (Brooks et al., 2009; So et al., 2011; Sowa et al., 2015). However, brood sizes were similar between *HB101* and *OP50* fed worms (Sowa et al., 2015). Thus, all life history trait experiments were performed using *HB101*. First, we analyzed *sphk-1(ok1097)/sphingosine kinase* mutants which contain a deletion in the kinase domain necessary for S1P production (Pitson et al., 2000; Chan et al., 2012). We found that *sphk-1* mutants have decreased lifespans, brood sizes and growth compared to wild type animals (Figures 2A–C and Table 2). Mean lifespans (average day of death) of *sphk-1* worms were significantly reduced compared to wild type animals (Figure 2A and Table 2). Mutants lacking *sphk-1* also exhibited reduced brood sizes (measured as the number of surviving progeny) compared to wild type animals (*p* < 0.001; Figure 2B and Table 2). After 48 h from egg lay, 31% of wild type animals developed to adult stage (young adult and adult), whereas only 9% of *sphk-1* mutants reached adulthood (Figure 2C and Table 2), suggesting the *sphk-1* mutants had a slower rate of development. Furthermore, the body lengths of *sphk-1* mutants were 12 and 24% smaller compared to wild type animals at 48 and 72 h, respectively (Table 2).

**Table 1:** Sphingolipid metabolic enzymes.

**Gene product**	**Worm ortholog**	**Alleles used**	**Known expression patterns**	**Human homolog**	**Protein similarity (%)**
Sphingosine kinase	*sphk-1*	*ok1097*	Neuronal	Sphk1	72.5
			Hypodermis		
			Intestine		
			BW Muscle		
Ceramide synthase	*hyl-1*	*gk203*	Ph Muscle	Ceramide synthase 6	71.9
			Intestine		
			Neuronal		
Ceramide synthase	*hyl-2*	*ok1766*	Ph Muscle	Ceramide sythase 4	67.7
			Intestine		
			Neuronal		
			Hypodermis		
Ceramide kinase	*cerk-1*	ok1252		Cerk	70.9

*BW, Body Wall; Ph, Pharynx*.

**Table 2:** Life history traits of sphingolipid metabolic enzyme mutants.

	**Lifespan (days)^∧^ number (censored animals) and trials indicated**	**Brood size (surviving progeny)**	**Development (48 h)**		**Development (72 h)**	
			**Body length (μm)**	**Percent adult (YA+A)**	**Body length (μm)**	**Percent adult (YA+A)**
wild type (*N2*)	20.1 ± 0.4 *n* = 90 (2), 4 trials	267.0 ± 18.6 *n* = 7	756.4 ± 11.5 *n* = 134	31.3 *n* = 303	1162.9 ± 12.5 *n* = 114	100 *n* = 386
*sphk-1 (ok1097)/ SPH kinase*	13.6 ± 0.4^**^n = 111 (3), 6 trials	88.2 ± 13.5^**^n = 5	666.9 ± 9.9^*^n = 38	9.2 *n* = 153	885.6 ± 12.3^**^n = 38	95.3 *n* = 191
*hyl-1(gk203)/ CER synthase*	16.13 ± 0.4^**^n = 100 (6), 4 trials	291.4 ± 28.0 *n* = 5	996.2 ± 13.4^**^n = 50	18.3 *n* = 104	1195.9 ± 12.2 *n* = 50	94 *n* = 116
*hyl-2(gnv1)/ CER synthase*	17.7 ± 0.6^**^n = 73 (2), 3 trials	248.5 ± 30.0 *n* = 10	792.3 ± 17.6 *n* = 90	2.6 *n* = 116	1152.2 ± 11.7 *n* = 86	89.1 *n* = 55
*sphk-1; hyl-1*	12.4 ± 0.4^**^n = 65 (6), 4 trials	104.4 ± 9.5^**^n = 5	662.7 ± 11.7^**^n = 41	0 *n* = 79	851.6 ± 14.1^**^ n = 58	78.9 *n* = 142
*sphk-1; hyl-2*	7.1 ± 0.7^**^n = 20 (0), 2 trials	9.7 ± 4.5^**^n = 8	647.9 ± 19.3^**^ n = 28	n/a	778.7 ± 26.1^**^ n = 27	n/a
*cerk-1(ok1252*)/ CER kinase	19.9 ± 0.3 *n* = 127 (2), 4 trials	244.7 ± 24.2 *n* = 9	760.7 ± 10.3 *n* = 84	34.1 *n* = 225	1045.9 ± 8.8^**^n = 32	100.0 *n* = 171

∧ *p-values determined by a Kaplan-Meier test and Bonferroni correction with the program OASIS (Yang et al., 2011)*.

* p < 0.01 and

** *p < 0.001, ANOVA and Tukey HSD compared to wild type and (± SEM for all data)*.

Previously, *sphk-1(ok1097)* worms analyzed for sphingolipid content by mass spectrometry exhibited increased levels of sphingosine and sphinganine (Menuz et al., 2009). This suggests that in *sphk-1* mutants, sphingosine cannot be converted to S1P. It should be noted, however, that S1P levels could not be measured due to the challenge of detecting the soluble lipid from worm extracts (Menuz et al., 2009). Thus, phenotypic differences observed in *sphk-1* mutants may be attributed to either increased SPH or decreased S1P. To differentiate between these possibilities, we used genetic manipulations that alter genes of sphingolipid metabolism enzymes acting in the sphingolipid salvage pathway.

The sphingolipid metabolic enzyme ceramide synthase converts SPH to CER, and mutants with mutations in ceramide synthase have increased SPH (Menuz et al., 2009). *C. elegans* have three ceramide synthase paralogs, *hyl-1, hyl-2*, and *lagr-1*. We examined *hyl-1(gk203)* and *hyl-2(gnv1)* mutants because these animals have reported increased SPH content, as determined by mass spectrometry (Menuz et al., 2009). Surprisingly, we found that both *hyl-1* and *hyl-2* mutants had decreased lifespans compared to wild type animals (Figure 2A and Table 2); however, these mutants had normal brood sizes compared to wild type animals (Figure 2B and Table 2). For developmental growth, we observed at 48 h that only 18% of *hyl-1/CER synthase* mutants reached adulthood and 2.6% of *hyl-2/CER synthase* mutants reached adulthood (Figure 2C and Table 2). This indicates that loss of ceramide synthase in *hyl-1* and *hyl-2* mutants may slow larval development compared to wild type animals. However, by 72 h, most *hyl-1* and *hyl-2* mutants reached adult stage.

To further examine the genetic interactions between the enzymes that regulate SPH and S1P metabolism, we examined *sphk-1;hyl-1* and *sphk-1;hyl-2* double mutants. The prediction is that the genetic interactions in these mutants lead to elevated SPH, which cannot be converted to S1P. Double mutants for *sphk-1;hyl-1* had shorter lifespans and smaller brood sizes than *sphk-1* mutants; furthermore, we did not observe any *sphk-1;hyl-1* double mutant animals reaching adulthood at 48 h (Figures 2A–C and Table 2). Intriguingly, *sphk-1;hyl-2* double mutants exhibited extremely poor health, making many analyses of phenotypes in these mutants difficult. However, these mutants had shorter maximum lifespans than *sphk-1* mutants alone and had an average of 9.7 ± 4.5 progeny per adult. Together, these data suggest that manipulations of sphingolipid metabolic enzymes alter development and shorten animal lifespan.

### Sphingosine kinase is necessary to slow the decline in locomotor activity of aged animals

**Figure 3:**
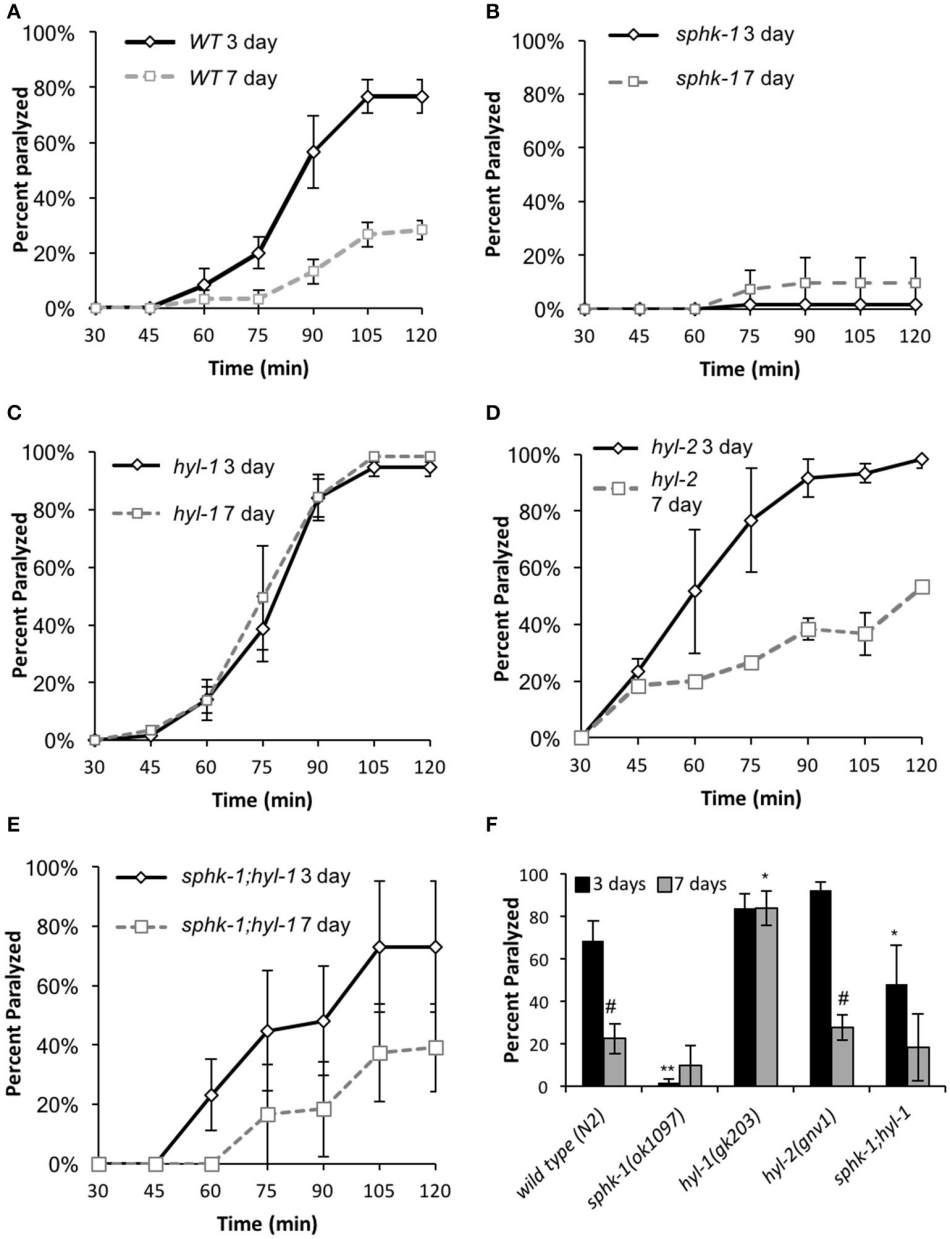
Sphingolipid enzymes regulate neuromuscular function. **(A–E)** Age-dependent changes in paralysis response of 3-day and 7 day old wild type animals to aldicarb (1 mM) exposure in the indicated strains. **(F)** Percent of animals paralyzed for the indicated strains after 90-min of aldicarb exposure. For all experiments, animals were examined 3 or 7 days post-hatching, and were transferred onto NGM plates containing aldicarb for testing. For all, error bars are ± SEM and are performed in triplicates; ^*^p < 0.01 and ^**^p < 0.001 for comparisons to the wild type counterpart and #*p* < 0.01 for comparisons to the 3 day old counterpart.

Sphingosine kinase recruitment to pre-synaptic release sites facilitates acetylcholine (ACh) release at neuromuscular and central synapses (Kanno et al., 2010; Chan et al., 2012). Thus, we aimed to determine whether neuromuscular function depended on (1) age, (2) sphingolipid signaling and (3) interactions between age and sphingolipids. First, we examined neuromuscular function by exposing wild type and sphingolipid mutants to an acetylcholine esterase inhibitor, aldicarb. Aldicarb inhibits the breakdown of ACh, resulting in muscle paralysis by hyper-contraction. On this pharmacokinetic agent, animals that have decreased ACh release paralyze at a slower rate compared to animals with normal signaling. We found that 7 day old wild type animals paralyzed slower than 3 day old wild type animals (ages are relative to egg lay, Figure 1B) (Figures 3A,F and Table 3, *p* < 0.01, ANOVA and Tukey's *post-hoc* test), suggesting that neuromuscular function is decreased in aged adult animals compared to younger adult animals.

**Table 3:** Values of survival to locomotor capacities and heat stress of all genotypes and ages.

	**Paralysis on aldicarb (% at 90 min timepoint)^∧^**		**Thrashing (per min)**		**Body bends (per min)**		**Heat stress survival (%, 6 h timepoint)**			**Heat shock survival (%)**		
	**3 day**	**7 day**	**3 day**	**7 day**	**3 day**	**7 day**	**3 days**	**7 days**	**11 days**	**3 days**	**7 days**	**11 days**
wild type (*N2*)	76.7 ± 8.7	13.3 ± 4.4#	197.3±.9.3	152.8 ± 12.4#	42.6±.1.5	25.3 ± 1.5#	62.0 ± 2.1	79.6 ± 1.3#	46.1 ± 1.3#	92.1 ± 2.5	96.7 ± 2.8	32.8 ± 2.5#
*sphk-1 (ok1097)/ SPH kinase*	1.7 ± 1.7^**^	9.5 ± 9.5	113.6 ± 7.5^*^	39.5 ± 8.3^*^#	31.7 ± 1.9^**^	10.6 ± 1.2^**^#	20.6 ± 1.9^**^	18.6 ± 1.7^**^	15.9 ± 7.4^**^	51.9 ± 1.6^**^	37.3 ± 2.8^**^#	6.9 ± 2.7^**^#
*hyl-1(gk203)/ CER synthase*	84.1 ± 6.6	84.3 ± 8.0^*^	212.3 ± 6.0	198.9±.10.6^*^	42.2 ± 2.6	33.8 ± 1.5	71.7 ± 1.6	86.3 ± 0.2	80.8 ± 1.1^**^	95.0 ± 4.8	93.6 ± 2.5	37.7 ± 2.5#
*hyl-2(gnv1)/ CER synthase*	91.7 ± 4.4	38.3 ± 7.2^*^#	203.4 ± 5.8	154.4±.12.6#	55.7 ± 2.4	26.9 ± 1.3	20.9 ± 2.9^**^	20.4 ± 3.2^**^	24.1 ± 3.6^**^	74.0 ± 5.2^*^	85.2 ± 0.4^*^	17.2 ± 2.4^**^#
*sphk-1/ hyl-1*	48.1 ± 18.4^*^	37.4 ± 16.3#	148.3 ± 9.9^*^	107.4±.8.3^*^	41.4 ± 1.9	26.2 ± 1.9	27.6 ± 2.7^**^	21.8 ± 1.4^**^	12.0 ± 3.1^**^	84.7 ± 0.5	89.6 ± 0.3^*^	22.5 ± 3.4^*^#
*sphk-1/ hyl-2*	33.0 ± 7.5	*n*/*a*										
*sphk-1; sphk-1 cDNA rescue*					38.0 ± 1.6	27.7 ± 2.1	45.0 ± 2.0^*^	76.3 ± 4.1#	48.4 ± 2.4	91.8 ± 1.6	93.4 ± 1.7	38.3 ± 3.3#
*cerk-1(ok1252)/ CER kinase*			204.8 ± 8.3	103.0 ± 12.9^*^#	44.4 ± 1.6	22.0 ± 1.5	68.8 ± 4.4	78.3 ± 1.5	36.9 ± 2.6			

∧ p-values determined by a Kaplan-Meier test and Bonferroni correction with the program OASIS (Yang et al., 2011). For all, data are ±SEM;

* p < 0.01 and

** p < 0.001 compared to wild type of the same age; chi-square and Log-Rank test were performed for heat stress analysis to get mean hours, and ANOVA and Tukey HSD tests were performed for all other experiments between genotypes.

# *p < 0.01, ANOVA and Tukey HSD were used to compare animals to the 3 day counterpart of the same strain*.

To compare between age and genotypes, we quantified animal paralysis after a 90-min exposure to aldicarb (Figure 3). We chose the 90-min time point because this is when approximately half of the wild types paralyzed. We found that mutations in *sphk-1* caused less paralysis compared to wild type animals (*p* < 0.001, Figures 3B,F and Table 3), consistent with previous findings (Chan et al., 2012). However, 3 day old mutants for two ceramide synthases, *hyl-1* and *hyl-2*, showed non-significant increases in their rates of aldicarb-induced paralysis at 90 min compared wild type animals (Figures 3C,D,F and Table 3); however, *hyl-1* ceramide synthase mutants were affected by mutations in *sphk-1* (*p* < 0.01, Figures 3E,F and Table 3), suggesting that increased neuromuscular function may be due to the conversion of SPH to S1P, as previously suggested (Chan et al., 2012).

Locomotor behavior and acetylcholine signaling have been shown to decline with age (Muir, 1997), so we next tested for an interaction between age-related loss of neuromuscular function and sphingolipid metabolism enzymes. To better compare across age, we again examined the 90-min time point in the aldicarb assay. In general, age decreased neuromuscular function of all genotypes examined [ANOVA, *F*_(1, 32)_ = 38.4, *p* = 0.001], supporting previous findings on age-related decrease in pharyngeal pumping (Mulcahy et al., 2012). In particular, wild type animals showed increased resistance to aldicarb from 3 to 7 days (*p* < 0.01), indicating that neuromuscular function declines with age (Figure 3F and Table 3). However, *hyl-1* and *hyl-2* ceramide synthase mutants, both of which paralyzed similarly to wild type at 3 days, demonstrated opposite responses with age. Specifically, the rates of *hyl-1* paralysis did not change with age. Seven-day old *hyl-2*, on the other hand, did show less paralysis than their 3-day counterparts, suggesting loss of neuromuscular function with age. However, 7 day paralysis of *hyl-2* mutants is still greater than wild type animals. Together, these data suggest that ceramide synthase mutations slow the observed decline of neuromuscular function in this paralysis assay. Interestingly, gene reporter experiments showed that *hyl-1* is expressed in neurons of worms, indicating that *hyl-1* may be the relevant CER synthase functioning in neurons (Chan and Sieburth, 2012). Similar to 3 day old animals, the effect of *hyl-1*/*ceramide synthase* mutations on slowing neuromuscular function decline of 7 day old animals was dependent on *sphk-1* (Figures 3E,F and Table 3). Thus, it is plausible that the phenotype observed in *hyl-1*/*ceramide synthase* may be due to the ability to convert SPH to S1P.

**Figure 4:**
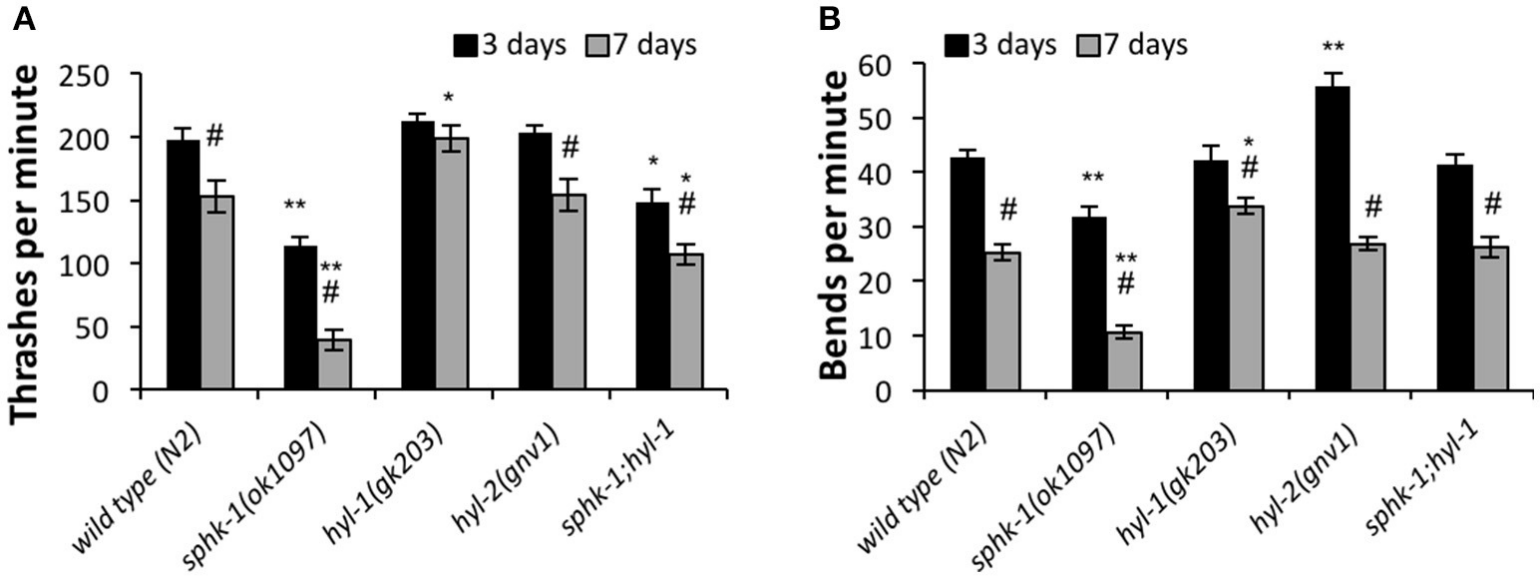
Sphingolipid enzymes regulate locomotor behavior. The number of thrashes per minute **(A)** and body bends per minute **(B)** for wild type and the indicated mutants, analyzed at 3 and 7 days (*n* = 25 for all groups). For all experiments, animals were examined 3 or 7 days post-hatching, and were transferred onto normal NGM plates for testing. For all, error bars are ± SEM; ^*^p < 0.01 and ^**^p < 0.001 for comparisons to the wild type counterpart, and #*p* < 0.01 for comparisons to the 3 day old counterpart.

To further address whether sphingosine kinase mediates the locomotor capacities of young and old animals, we analyzed lateral movement of animals on liquid and solid medium (Figure 4). To measure movement in liquid medium, we performed a thrashing assay. We found that 3 day old *sphk-1* mutants demonstrated fewer thrashes per minute (tpm) than wild type animals (*p* < 0.01; Figure 4A and Table 3) and fewer bends per minute than wild type animals (*p* < 0.001; Table 3 and Figure 4B). Similar to paralysis on aldicarb, 3 day old *hyl-1* mutants showed similar thrashing and body bending counts as wild type animals. However, *sphk-1;hyl-1* double mutants showed less thrashing than wild type animals. Together, these data suggest that animals containing the *sphk-1* mutation leads to less movement capabilities compared to wild-type adult animals.

Similar to assays of neuromuscular function, age had a significant effect on declining locomotion in liquid medium [ANOVA, *F*_(1, 288)_ = 103.5, *p* < 0.001] and solid surfaces [ANOVA, *F*_(1, 288)_ = 326.4, *p* < 0.001]. Pairwise analyses showed that wild type worms had 22.5% less thrashes per minute at 3 days compared to 7 days (*p* < 0.01; Figure 4A and Table 3) and 40.7% less body bends at 3 days compared to 7 days (*p* < 0.001; Figure 4B and Table 3). Seven day old mutants lacking *sphk-1*, compared to 3 day counterparts, significantly decreased their thrashing behavior by 65.2% and their bending by 66.5%; Seven day old *sphk-1* mutants also performed significantly less than 7 day wild type counterparts in both assays (Figure 4 and Table 3). Furthermore, expressing *sphk-1* under its endogenous promoter can rescue the loss of body bends behavior in 3 and 7 day old *sphk-1* mutants (Table 3). However, 7 day old *hyl-1* mutants did not show decline in thrashing or body bends when compared to their 3 day old counterparts, and conversely had increased thrashes (Figures 4A,B and Table 3). However, this thrashing phenotype was not observed in *sphk-1;hyl-1* double mutants (28% drop in thrashes from 7 to 3 days, *p* = 0.05). Together, our findings suggest that loss of sphingosine kinase may be lead to quicker locomotor activity decline with age.

### Ceramide kinase is not required for development and normal movement

The sphingolipid metabolic enzyme ceramide kinase is found in synaptic vesicles preparations, and knockouts of CER kinase in flies and mice produce neuronal defects (Bajjalieh et al., 1989; Bornancin, 2011). Thus, we aimed to determine whether loss of CER kinase was important for lifespan and maintaining locomotor function. The *C. elegans* CER kinase (*cerk-1*) is orthologous to the human CERK, with 70.9% similarity in amino acid sequence (Table 1). It also contains a conserved DAG kinase domain and a conserved cysteinyl motif (CX_3_CX_2_C) in the regulatory loop, both of which are essential for mammalian CERK function (Bornancin, 2011). We obtained the allele (*ok1252*) and identified a 570 bp deletion in this allele, which leads to a 190 amino acid deletion, spanning most of the conserved DAG kinase domain of ceramide kinase. We did not detect differences in lifespan, brood size, or 2-day growth compared to wild type animals (Table 2). However, there was a small but significant decrease in body length after 72 h of development.

To determine whether loss of *cerk-1/CER kinase* altered movement, we examined mutants in thrashing and body bending assays. At 3 days old (relative to egg lay), *cerk-1* mutants exhibited similar thrashing and body bends behavior compared to wild type animals. However, *cerk-1* mutants had a 49.7% decrease in thrashing at 7 days (*p* < 0.01 compared to the wild type 7 day counterpart) but not bending on solid medium. Together, this suggests that loss of CER kinase does not alter movement at young adult ages, but may lead to a faster decline in movement in liquid medium.

### Sphingolipid metabolic enzymes regulate stress response in *C. elegans*

**Figure 5:**
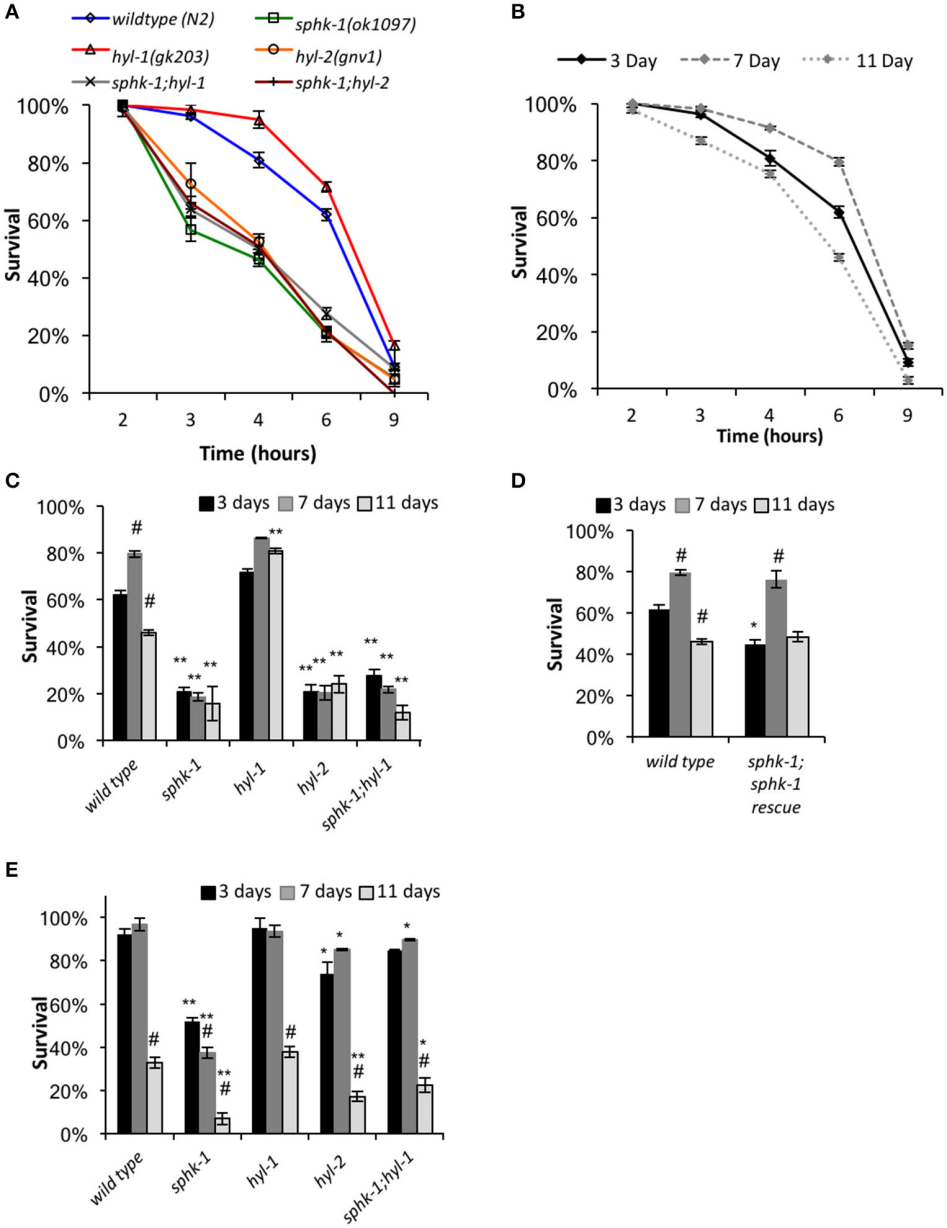
Response to heat stress is influenced by sphingolipid enzymes and age. **(A)** Chronic heat stress assay on the indicated animals. Wild type and mutant animals were exposed to 35°C (from room temperature) and the fraction of survival was tallied at the times indicated. **(B)** Wild type animals of different ages responded differently to heat stress. **(C,D)** Fraction of survival of wild type and indicated mutants at 3, 7, or 11 days old exposed to 35°C at the 6 h time point. **(E)** Response of animals to an acute heat shock assay (37°C for 90 min). Fraction of survival of wild type and indicated mutants is shown at 3, 7, or 11 days old. For all, error bars are ± SEM, ^*^p < 0.01 and ^**^p < 0.001 for comparisons to the wild type counterpart, and #*p* < 0.01 for comparisons to the 3 day old counterpart.

When exposed to stressors, neurons and other cells mobilize defenses by activating critical signaling pathways important to cell survival, including sphingolipid signaling. Thus, alterations of sphingolipid enzyme activity within cells may impact an animal's ability to tolerate stress. To further analyze this, we examined the survival of 3-day old animals (relative to egg lay) subjected to prolonged heat (35°C) measured over a 9-h period. We found that as the duration of heat exposure increased, survivability of animals decreased, and the majority of animals of all the genotypes tested were unresponsive to prodding by 9 h (Figure 5A and Table 3).

Mutants lacking *sphk-1* showed less resistance to death induced by heat compared to wild type animals at 3 days old (χ^2^ = 33.2, *p* < 0.001, Log-Rank Test; Figure 5A and Table 3). Expressing *sphk-1* under its endogenous promoter partially restored *sphk-1* survival near wild type survival levels, suggesting a functional rescue (Figure 5D and Table 3). On the other hand, *hyl-1* mutants were as resistant to heat stress as wild type animals at the 6 h time point but more resistant over the course of the 9 h exposure (χ^2^ = 4.4, *p* < 0.05, Log-Rank Test; Figures 3A,C and Table 3). Previous studies of other CER synthase mutations showed that double mutants for *hyl-1(ok976);lagr-1(gk331)* had resistance to heat stress, which was most evident at more than 8 h of exposure (Mosbech et al., 2013). Interestingly, *hyl-2*/CER synthase mutants were much more sensitive to heat stress than *hyl-1* mutants (χ^2^ = 40.0, *p* < 0.001, Log-Rank Test), suggesting that the two genes may have varied functions. This corroborates previous work showing that *hyl-2* mutants are more susceptible to anoxic stress than *hyl-1* mutants (Menuz et al., 2009).

The finding that *sphk-1* mutants had decreased survival during heat stress could be due to increased SPH or decreased S1P levels. Because *hyl-1* mutants have increased SPH levels (Menuz et al., 2009) and demonstrated enhanced survival during prolonged heat exposure, poor heat stress response might be due to decreased ability to convert SPH to S1P. To further confirm this genetically, we examined heat stress response in double mutant animals lacking both *sphk-1 and hyl-1 (sphk-1;hyl-1)*. We found that *sphk-1;hyl-1* responded similarly to heat exposure as *sphk-1* mutants alone (χ^2^ = 0.9, *p* > 0.05, Log-Rank Test; Figure 5A and Table 3), suggesting that the *sphk-1* mutation contributes to poor heat stress response. In addition, *sphk-1;hyl-2* double mutants also exhibited a reduced ability to resist heat stress similar to either *sphk-1* or *hyl-2* mutants alone.

### Heat stress responses of aged animals depends on sphingosine kinase

As animals age, their ability to resist stress decreases, accelerating age-related and stress-dependent decline in critical cellular functions. Sphingolipid metabolism alters with age and may affect an organism's ability to withstand temperature changes. Thus, we next examined whether sphingolipid manipulations altered the survival of aged animals to heat exposure. We tested animals of all genotypes at ages of 3 days (start of adult stage), 7 days (a period when reproduction has mostly passed), and 11 days of age (a period when animals display noticeable age-related changes in physiology). All ages are relative to egg lay (Figure 1B). Seven-day old wild type animals showed a resistance to heat stress when chronically exposed to 35°C compared to 3 day old animals (mean survival is 7.4 ± 0.1 h at 3 days vs. 8.2 ± 0.2 at 7 days, χ^2^ = 8.35, *p* < 0.01, Log-Rank Test), whereas 11 day old animals exhibited reduced heat stress resistance (mean survival was 6.7 ± 0.2, Figures 5A,B) compared to 3 day and 7 day old animals (χ^2^ = 14.2 and 30.0, respectively; *p* < 0.01 for both).

To better compare animals across genotype and age (3, 7, and 11 day old animals), we analyzed animal survival to prolonged heat stress at the 6 h time point of exposure to 35°C. This time was used because it represents a time at which approximately 50% of wild type animals had died. For wild type animals, the percent survival at 3 days was 62.0 ± 2.1%, which increased to 79.6 ± 1.3% at 7 days (*p* < 0.01, ANOVA and Tukey's *post-hoc* Tests), and decreased to 46.0 ± 1.3% at 11 days (*p* < 0.01, Figures 5C,D and Table 3). Sphingolipid mutants demonstrated more variable patterns than wild type. *sphk-1* mutants showed poor heat stress responses at all ages compared to wild type (*p* < 0.001 for all), and we did not detect differences between ages. The resistance of wild type animals to heat stress at 7 days compared to 3 days may be due to FUdR treatment alone, as previous studies have shown that FUdR increases heat stress resistance, albeit at 8x higher concentrations than that used here (Feldman et al., 2014). However, our data indicate that *sphk-1* mutants are likely resistant to FUdR mediated effects. In addition, 3 day old *sphk-1* mutants pre-treated to 24 h of FUdR (50 μM) prior to the heat stress assay also did not improve heat stress resistance compared to non-FUdR treated mutants (data not shown).

Both *sphk-1(ok1097)* and *hyl-1(gk203)* mutants have previously been found to have elevated SPH levels (Menuz et al., 2009). Whereas, *sphk-1* mutants have less heat stress resistance than wild type animals, *hyl-1* mutants had greater survival to heat stress at 11 days compared to wild type counterparts (*p* < 0.001). To determine whether the phenotype observed in *hyl-1* mutants depends on sphingosine kinase, we examined *sphk-1;hyl-1* double mutants. We found that their heat stress resistance at 11 days was less than wild type animals (Figure 5C and Table 3, *p* < 0.001, ANOVA and Tukey's *post-hoc* Tests). This suggests that the effect of *hyl-1* single mutants on heat stress resistance depends on *sphk-1*.

Next, we examined the sphingolipid pathway for a role in acute responses to heat stress. For this, animals were subjected to an acute heat exposure at 37°C for 1.5 h, and then measured for survival after 12 h. We found that *sphk-1* mutants had a larger drop in survival from 7 to 11 days compared to wild type animals. Specifically, wild type animals had a 92.1 ± 2.5% survival at 3 days, which stayed high at 7 days (96.7 ± 2.8% survival) but dropped at 11 days (32.8 ± 2.5% survival) (Figure 5E and Table 3). From 7 to 11 days, there was a 66% decrease in the number of surviving animals. Mutants of *sphk-1* demonstrated reduced survival at 7 days (37.3 ± 2.8%, 28% loss in response from 3 days) and at 11 days (6.9 ± 2.7% survival, which was an 82% loss of response from 7 days). This large drop in heat shock survival from 7 to 11 days was also found in *sphk-1;hyl-1* double mutants when compared (74% loss of response). Together, these findings suggest that loss of sphingosine kinase may accelerate death following stress exposure, and that S1P production may be important for promoting stress resistance in aged animals.

## Discussion

### The functions of sphingosine kinase on reproduction, growth, and lifespan

We found that genetic manipulations in *C. elegans* enzymes that affect the ability to convert the sphingolipid sphingosine to S1P, including *sphk-1* single mutants and mutants lacking both *sphk-1* and ceramide synthase (*sphk-1*;*hyl-1* and *sphk-1;hyl-2)*, resulted in significantly reduced lifespans compared with wild type animals (summarized in Table 4). The effect of gene mutations in sphingolipid enzymes on lifespan may be accounted for by the loss of enzymatic activity, yielding elevated sphingosine and/or ceramide, decreased S1P, or both. Indeed, loss of cellular levels of S1P contribute to aging phenotypes. For example, reduced S1P levels are observed in pathologies associated with Alzheimer's disease (He et al., 2010). In addition, S1P can bind and stabilize human telomerase reverse transcriptase (hTERT), the catalytic subunit of the enzyme telomerase (Panneer Selvam et al., 2015). Given that telomerase maintains telomere length of chromosomes, loss of S1P may alter the functions of telomerase, leading to telomere shortening and accelerated cell senescence and aging.

**Table 4:** Lifespan and stress response phenotypes of sphingolipid mutants.

**Gene product**	**Worm ortholog**	**Alleles**	**Lifespan phenotype % compared to wildtype (references)**	**Stress response (references)**
Sphingosine kinase	*sphk-1*	*ok1097*	54 (Table 2)	Hypersensitive to heat stress (Table 3)
		*ok1097*		No change to anoxia (Menuz et al., 2009)
		*ok1097*	80 (L1 diapause) (Cui et al., 2017)	
S1P lyase	*spl-1*	*n/a;* RNAi	78 (Samuelson et al., 2007)	
Ceramide synthase	*hyl-1*	*gk203*	80 (Table 2)	Resistance to heat stress (Table 3)
		*gk203*	117 (Menuz et al., 2009)	Resistance to anoxia (Menuz et al., 2009)
		*ok976*	no change (Menuz et al., 2009)	Resistance to anoxia (Menuz et al., 2009)
		*ok976*	67 (L1 diapause) (Cui et al., 2017)	
		*ok976*	no change^*^ (Tedesco et al., 2008)	
	*hyl-2*	*gnv1*	88 (Table 2)	Hypersensitive to heat stress (Table 3)
		*gnv1*	no change (Menuz et al., 2009)	Hypersensitive to anoxia (Menuz et al., 2009)
		*ok1766*	78 (L1 diapause) (Cui et al., 2017)	
		*tm2031*	no change (Menuz et al., 2009)	Hypersensitive to anoxia (Menuz et al., 2009)
	*larg-1*	*gk327*	no change (Cui et al., 2017)	
	*hyl-1;lagr-1*	*ok976;gk327*	18 (Cui et al., 2017)	
		*hyl-1;lagr-1*	121 (Mosbech et al., 2013)	Resistance to heat shock (Mosbech et al., 2013)
	*hyl-2;lagr-1*	*ok1766;gk327*	80 (L1 diapause) (Cui et al., 2017)	
Ceramide kinase	*cerk-1*	*ok1252*	no change (Table 2)	
acid sphingo-myelinase	*asm-3*	*ok1744*	114 (Kim and Sun, 2012)	
	*Neutral sphingo-myelinase*	n/a; G109 (drug)	106 (Cutler et al., 2014)	
serine palmitoyl-transferase	*sptl-1*	*ok1693*	135 (Cutler et al., 2014)	
	*sptl-2*	*ok2753*	34 (Cui et al., 2017)	
	*sptl-3*	*ok1927*	88 (Cui et al., 2017)	
	serine palmitoyl-transferase	n/a; ISP-1 (drug)	131 (Cutler et al., 2014)	

* *hyl-1(ok976) mutants originally showed an increased lifespan phenotype, but subsequent outcrossing by Tedesco et al. indicated that backcrossed strains showed normal phenotypes*.

Several lines of evidence also support the theory that manipulation of enzymes that elevate ceramide decrease lifespan (Table 4). In worms, mutations in or RNAi knockdown of acid sphingomyelinase (*asm-3*) increase lifespan (Kim and Sun, 2012), suggesting that reduced ceramide production is correlated to longevity. In addition, 11 day old worms accumulate sphingolipids, particularly glucosylceramides (GM1 and GM3) and specific sphingomyelins, compared to 3 day old counterparts (Cutler et al., 2014). Genetic or pharmacological inhibition of serine palmitoyl transferase, an upstream enzyme in sphingolipid metabolism, and glucosylceramide synthase, an enzyme important for producing glucosylceramides, decreased respective lipid levels, slowed development and increased lifespan (Cutler et al., 2014). Indeed, in non-mammalian genetic model organisms, such as yeast cells, deletion of ceramide synthases increase replicative lifespan (the number of times cells can bud), but not chronological lifespan (survival after cells have stopped dividing) (Huang et al., 2014). However, manipulations of other sphingolipid metabolism enzymes, such as YDC1, a yeast ceramidase gene, decrease chronological lifespan. Together, these findings suggest that mobilization of ceramide and sphingosine production may decrease lifespan. However, our data suggests that the inability to convert sphingosine to S1P may worsen lifespan phenotypes, in addition to age-related decline in movement and stress response.

Ceramides can get converted into other sphingolipids such as sphingosine, which may also affect lifespan. However, genetic manipulations of enzymes acting on the lipid sphingosine have yielded conflicting results on the effects of sphingolipids on lifespan (Table 4). For example, the effect of ceramide synthase mutations on lifespan appears to depend on dose and genetic background. Double mutants of ceramide synthase (*hyl-1(ok976);lagr-1(gk327)*) have extended lifespan (Mosbech et al., 2013). Thus, we were surprised that the *hyl-1* and *hyl-2* single mutants examined in this study showed reduced lifespan; however, consistent with our findings, *hyl-1* and *hyl-2* mutants showed reduced longevity in starvation-induced L1 diapause animals (Cui et al., 2017). Furthermore, data here and results from others have shown that the *hyl-1(gk203)* allele does not lead to extended lifespan (Tedesco et al., 2008; Menuz et al., 2009; Cui et al., 2017). In addition, Tedesco et al. (2008) also report that initial analyses of *hyl-1(ok976)* mutants showed increased lifespan, but strains backcrossed (greater than 6x) no longer exhibited long-lived phenotypes compared to their wild type strain. Thus, *hyl-1* strains of allele *ok976* may have linked mutations that affect lifespan (Tedesco et al., 2008; Menuz et al., 2009). The *hyl-1(gk203)* strain used here was outcrossed 10x previously (Chan et al., 2012). Furthermore, Tedesco et al. (2008) showed that first generation RNAi knockdown of the lag1p motif of *hyl-1* resulted in increased lifespan, but no lifespan difference was observed in second or third generation RNAi treated animals. Another explanation for differences in our experiments is that our lifespan experiments were performed at RT, which may have impacts on the fluidity or composition of the lipid membrane. Indeed, disrupting sphingolipids with myriocin, an inhibitor of serine-palmitoyl transferase (SPT) that depletes sphingolipids, in *S. cerevisiae* altered membrane order by reducing the amount of microdomains present (Vecer et al., 2014). Interestingly, pharmacological inhibition of SPT increases lifespan (Table 4), and others have suggested that altering sphingolipid domains may alter insulin signaling (Holland et al., 2007; Holland and Summers, 2008; Kim and Sun, 2012; Cutler et al., 2014). Thus, temperature dependent changes in sphingolipids may affect lifespan and physiological phenotype. Nonetheless, subsequent experiments, including body bends, thrashing, and aldicarb experiments, performed on worms grown at 20°C have shown that *sphk-1* loss of function or knockdown by RNAi show that *sphk-1* decreases neuromuscular function as seen at room temperature (data not shown). Furthermore, Cui et al. (2017) showed that *sphk-1, hyl-1* and *hyl-2* mutants had decreased survival during L1 diapause when grown at 20°C (summarized in Table 4).

Sphingolipids can be composed of shorter or longer acyl-chain fatty acids. Interestingly, mutations in ceramide synthases genes, whose proteins act on shorter acyl-chain sphingosine (< 22 carbons), decreased lifespan, whereas mutations in ceramide synthases acting on longer sphingosine did not (Mosbech et al., 2013). However, longer acyl chain lipids promote survival under starvation conditions (Cui et al., 2017). Thus, the impact of genetic modifications on lipid levels, the complexity of the sphingolipid modification, and strain differences likely led to discrepancies on the effects of ceramide synthase mutants on lifespan.

Sphingolipids are a complex class of lipids, and lipid molecules can be made via salvage, *de novo*, or recycling pathways, which differ in their cellular source of sphingosine backbone. *De novo* pathways generate ceramides from the activity of the enzyme serine palmitoyltransferase (SPT); salvage pathways generate ceramide molecules from endocytosed lipids and complex sphingolipids such as glucosylceramides and sphingomyelin; and recycling pathways from the activity of recycling enzymes (Hannun and Obeid, 2008; Huang et al., 2014). In addition, sphingolipids can be modified by addition of sugar moieties or a change in carbon chain length. These complex metabolites have varied effects on animal growth and lifespan. Loss of function mutations in *C. elegans* SPT gene, *sptl-1*, or pharmacological inhibition of glucosylceramide synthases increased lifespan (Cutler et al., 2014). Thus, it is apparent that the roles of sphingolipids in regulating lifespan are not simple, and is likely due to combinatory effects of altered sphingolipid enzyme activity in various cellular pathways of animals.

We also found that loss of sphingosine kinase leads to a reduced brood size, whereas genetic manipulations in ceramide synthase have normal brood sizes. S1P has previously been shown to be critical for gonad cell function (Guo et al., 2014); S1P can protect from germline oocyte apoptosis in mice, and reduced S1P in fly *Sk2* mutants had 43% of the progeny as wild type controls (Morita et al., 2000; Herr et al., 2004). Furthermore, *sphk-1* and *sphk-1;hyl-1* double mutants had a slightly decreased development and body size compared with wild type, suggesting enzymes in sphingolipid metabolism may alter the rate at which an organism develops. Taken together, our data suggest that sphingolipids are critical during both reproduction, development and longevity, and that genetic manipulations of sphingolipid metabolism that lower S1P levels are likely to accelerate aging and reduce lifespan.

The interaction of sphingolipids with the *daf-2/insulin like growth factor 1 receptor* signaling pathway (IIS) is also unclear. Several lines of evidence suggest that sphingolipid enzymes act parallel to *daf-2* signaling pathways. For example, RNAi knockdown of *daf-2* in *hyl-1(ok976);lagr-1(gk327)* mutants had additive effects (Mosbech et al., 2013). CER synthases have also been shown to act independently of the *daf-2* signaling pathway in stress responses; *hyl-2* was also shown to mediate responses to anoxia, which are independent of *daf-2* (Menuz et al., 2009) and *hyl-1;lagr-1;age-1* triple mutants had intermediate responses to starvation induced D1 diapause survival compared to *hyl-1;lagr-1* and *age-1* alone (Cui et al., 2017). On the other hand, mutations in *asm-3*/*acid sphingomyelinase* lead to a modest increase in lifespan, and the phenotype is partially dependent on the insulin receptor *daf-2* signaling (Kim and Sun, 2012)*;* the authors found that *asm-3* mutations did not further increase *daf-2* lifespan nor alter the decreased *daf-16* lifespan phenotype, and propose a model where an altered the sphingolipid membrane and lipid raft environment may alter receptor localization or function (Kim and Sun, 2012). However, the interaction of sphingosine kinase with *daf-2* is unknown. Interestingly, RNAi knockdown of *spl-1*, the putative S1P lyase, did not increase lifespan as expected but rather reduced lifespan compared to wild-type and increased mortality in *daf-2* mutants (Samuelson et al., 2007). However, *spl-1* mutations and knockdown has been shown to cause developmental defects as well (Mendel et al., 2003; Samuelson et al., 2007). In mammalian tissues, S1P acts through S1P receptors to modulate insulin receptors and insulin resistance (Fayyaz et al., 2014). However, *C. elegans* do not appear to have S1P receptors orthologs.

### Sphingosine kinase helps maintain locomotor activity with aging

We show that loss of *sphk-1* decrease neuromuscular function, thrashing, and body bending, suggesting that S1P levels are critical to promoting neuromuscular function. Consistent with this, S1P promotes neurotransmitter release at central synapses and neuromuscular junctions (Okada et al., 2009; Chan et al., 2012). Furthermore, both fly and worm sphingosine kinase mutants have functional neuromuscular defects, but have no gross morphological defects and normal muscle responsiveness (Herr et al., 2004; Chan et al., 2012), suggesting that S1P is a mediator of neuronal function.

Aging has been associated with loss of neuronal function, including motor and cognitive decline. Here, we show that mutations in sphingosine kinase lead to greater decline of neuromuscular function of aged animals. Neuronal deficits during aging may stem from synapse deterioration, reduced neurotransmitter release, and neuronal loss among other neuronal malfunctions. It is possible that neurons undergoing aging and stress respond by recruiting sphingosine kinase to membranes during aging, as shown in tissues of young animals (Okada et al., 2009; Pitson, 2011; Chan et al., 2012). With aging, there could be a lower rate of recruitment of sphingolipid metabolic enzymes such as sphingosine kinase, leading to higher cellular levels of ceramide and sphingosine and lower levels of S1P, ultimately leading to decreased neuronal function. Indeed, inhibition of sphingosine kinase and sphingosine itself mediate hair cell loss in cochlear cells of rats, whereas S1P decreased hair cell loss (Romero-Guevara et al., 2015; Tani et al., 2016). Furthermore, regulation of S1P levels may impact neurodegenerative disorders. For example, inhibiting sphingosine kinase decreases dopaminergic neuron viability by increasing ROS, and Sphk1 expression is decreased in a Parkinson's disease model (Pyszko and Strosznajder, 2014). Moreover, lower levels of S1P are observed in tissues of Alzheimer's diseased patients and elevated expression of S1P lyase, which breaks down S1P, enhances cerebellar neurodegeneration (He et al., 2010; Van Echten-Deckert and Walter, 2012; Ceccom et al., 2014). It will be of considerable interest to better understand sphingosine kinase function in aging neurons.

Ceramide kinase, which produces ceramide-1-phosphate (C1P), is found in synaptic vesicle preparations, and C1P can stimulate neurotransmitter release (Bajjalieh et al., 1989; Bornancin, 2011). However, we found that loss of ceramide kinase in young adults did not affect motor performance in thrashing or body bending in young animals. Similarly, ceramide kinase-null mice did not exhibit differences in motor performance on rotorod tests, but did exhibit defects in emotional behavior in open field tests (Mitsutake et al., 2007). Interestingly, we found that *cerk-1*/CERK mutants exhibited a slight early decline in thrashing movement at older ages. Interestingly, C1P signaling can promote muscle regeneration (Gangoiti et al., 2012). Thus, these findings suggest that ceramide kinase loss may not be important for normal neuronal function, but may have modulatory roles in neurons or muscles exposed to stress or in aging conditions.

### Loss of sphingosine kinase affects stress responses and survival

The resilience of animals to exposure to stress may be a contributing factor to healthy aging. We observed that mutations in sphingosine kinase also decreased heat stress response, and effects were larger at older ages. *sphk-1* single mutants did appear less healthy overall, as poor stress response was observed even at young ages. Thus, developmental roles of sphingosine kinase may have contributed to poor responses. Intriguingly, *hyl-1*/ceramide synthase mutants had slightly shorter mean lifespans than wild type animals, but exhibited better responses at older ages to heat stress and age-dependent motor decline than wild type counterparts. Thus, these mutants may live a longer portion of their lifespan in a healthy state. These effects were blocked by genetic mutations in *sphk-1*. It is interesting to speculate that at young ages, elevated sphingosine can promote health by being converted to S1P, but at older ages (and in *sphk-1* mutants), less S1P is made and elevated SPH may lead to poor health. Previous studies linking lifespan to healthy aging, or healthspan, have shown that long lived *daf-2* mutants spend a smaller portion of their lifespan with better heat stress response and movement capacity (Bansal et al., 2015). However, this was not true for all longevity models and performance with age depended on the physiological measure. For example, maximum movement velocity was not dependent on lifespan, as *daf-2* and *daf-16* animals spent the same percent of their lifespan in movement performance as wild type animals (Hahm et al., 2015).

How might genetic manipulations affecting the production of S1P affect stress response and survival? S1P is known as a cell survival, stress response, and immune response factor, and increased S1P may protect cells against damage (Hait et al., 2006; Huang et al., 2014). In fact, blocking S1P breakdown increases cell viability in cultured cells and S1P can activate extracellular receptors that initiate cell survival signaling pathways such as MAP kinase, PI-3 kinase, and mTOR (Johnson et al., 2003; Taniguchi et al., 2012; Van Brocklyn and Williams, 2012). In neurons, blocking SPH to S1P conversion reduced the neuroprotective effect of the pro-survival factor docosahexaenoic acid (DHA). However, *C. elegans* and many invertebrates do not have homologs to mammalian S1P receptor genes, suggesting that non-receptor mediated functions of S1P may also mediate cell survival and longevity. For example, fibroblasts overexpressing sphingosine kinase, yet lacking S1P receptors, still have pro-survival effects (Olivera et al., 2003). Activating sphingosine kinase with K6PC-5 induces anti-aging effects through intracellular calcium (Youm et al., 2008). In addition, mammalian Sphk2 colocalizes with HDACs, and S1P is thought to inhibit the deacetylation of histones and alter transcription (Hait et al., 2009). Thus, S1P may have non-receptor dependent functions in stress response and aging. Together, sphingosine kinases may have myriad functions in the cells, but the mechanisms of sphingosine kinase in stress response and aging pathways are still unclear.

In both vertebrate and invertebrate models, stress activates signaling pathways that mobilize sphingosine kinase, increase S1P levels, and promote cell survival. For example, oxidative stress stimulates recruitment of SphK to membranes, where it may produce S1P (Maceyka et al., 2007; Van Brocklyn and Williams, 2012). In yeast, heat stress mobilizes sphingolipid metabolism, which may regulate p-body formation and translation of stress response genes (Cowart et al., 2010; Liu et al., 2013). Furthermore, growth factors such as TGFβ and cytokines activate mammalian SphK and increase S1P in cells (Alvarez et al., 2007). On the other hand, inhibiting SphK activity leads to increased ROS toxicity in tissues such as the liver, and increased ROS has also been shown to degrade SphK (Pchejetski et al., 2007; Chatzakos et al., 2012). Thus, sphingosine kinase activity is an important part of the stress response mechanism, and poor sphingolipid mobilization in response to stress conditions during aging may contribute to deteriorating health span of animals. In addition, stress from ultraviolet light may mobilize salvage pathways to increase sphingolipid metabolism in cells, and activation of PKC by phorbal esters may increase ceramide production via the salvage pathway in a ceramide synthase dependent manner to resist stress induced cell death (Kitatani et al., 2006, 2008). Here, we show that manipulations in sphingosine kinase in animals resulted in poor survival of animals exposed to acute heat shock compared to wild type counterparts, and this effect increased with older age.

Consistent with a role of the sphingolipid rheostat in survival, it is possible that cells balance S1P and ceramide production in response to varying levels of environmental stress. Under low levels of oxidative stress, cells are thought to mobilize a coordinated response involving the activation of sphingomyelinases and sphingosine kinases (Van Brocklyn and Williams, 2012). The net result is to increase ceramide in cells, but to convert CER/SPH to S1P under the regulation of sphingosine kinase. However, under extreme stress, cells may undergo regulated apoptosis and mobilize sphingolipid metabolism toward sphingosine and ceramide. Indeed, in *C. elegans*, ceramide recruitment to mitochondria is important for germ cell apoptosis when exposed to radiation, and ceramide production is important for mitochondrial stress response to bacterial pathogens, activation of detoxification genes, and survival during anoxia and radiation (Deng et al., 2008; Menuz et al., 2009; Liu et al., 2014). Furthermore, ceramide induced inflammatory responses through NFKappaB may mediate aging (Wu et al., 2007). Thus, aging may result in mobilization of cell signaling pathways that regulate enzymes in sphingolipid metabolism, particularly those affecting ceramide and S1P balance.

In conclusion, we identify that sphingolipid metabolic enzymes that affect the CER/SPH and S1P rheostat alter the responses of aged animals to neuromuscular function and stress. In older animals, losing the ability to produce S1P results in a loss of performance of parameters of healthy aging, including resilience to heat exposure and maintaining locomotor function. Recent studies on aging have tried to uncouple phenotypes associated with lifespan and health span (Keith et al., 2014; Bansal et al., 2015), and efforts to study specific cell signaling pathways that promote healthy aging is a global concern. Life expectancy has been increasing in the American (and global) population (according to the *Global Health and Aging Report*, National Institutes of Aging), and so have the incidences of age-related disorders. Given the increased loss of memory, cognitive function, and movement in the elderly, studies of sphingolipid signaling may lead to a greater understanding of mechanisms for healthy neuronal aging.

## Author contributions

JC conceived and performed experiments, analyzed and interpreted data, and composed the manuscript. DS, AN, JB, BH and HH performed experiments, analyzed data, and aided in the initial manuscript draft. TS performed experiments, composed the manuscript, and interpreted and analyzed data.

### Conflict of interest statement

The authors declare that the research was conducted in the absence of any commercial or financial relationships that could be construed as a potential conflict of interest.

## References

[B1] Alvarez S. E. Milstien S. Spiegel S. (2007). Autocrine and paracrine roles of sphingosine-1-phosphate. Trends Endocrinol. Metab. 18, 300–307. 10.1016/j.tem.2007.07.005 17904858

[B2] Bajjalieh S. M. Martin T. F. Floor E. (1989). Synaptic vesicle ceramide kinase. a calcium-stimulated lipid kinase that co-purifies with brain synaptic vesicles. J. Biol. Chem. 264, 14354–14360. 2547795

[B3] Bansal A. Zhu L. J. Yen K. Tissenbaum H. A. (2015). Uncoupling lifespan and healthspan in *Caenorhabditis elegans* longevity mutants. Proc. Natl. Acad. Sci. U.S.A. 112, E277–E286. 10.1073/pnas.1412192112 25561524 PMC4311797

[B4] Bornancin F. (2011). Ceramide kinase: the first decade. Cell. Signal. 23, 999–1008. 10.1016/j.cellsig.2010.11.012 21111813

[B5] Brailoiu E. Cooper R. L. Dun N. J. (2002). Sphingosine 1-phosphate enhances spontaneous transmitter release at the frog neuromuscular junction. Br. J. Pharmacol. 136, 1093–1097. 10.1038/sj.bjp.0704839 12163341 PMC1573457

[B6] Branicky R. Desjardins D. Liu J. L. Hekimi S. (2010). Lipid transport and signaling in *Caenorhabditis elegans*. Dev. Dyn. 239, 1365–1377. 10.1002/dvdy.22234 20151418

[B7] Brooks K. K. Liang B. Watts J. L. (2009). The influence of bacterial diet on fat storage in *C. elegans*. PLoS ONE 4:e7545. 10.1371/journal.pone.0007545 19844570 PMC2760100

[B8] Ceccom J. Loukh N. Lauwers-Cances V. Touriol C. Nicaise Y. Gentil C.. (2014). Reduced sphingosine kinase-1 and enhanced sphingosine 1-phosphate lyase expression demonstrate deregulated sphingosine 1-phosphate signaling in Alzheimer's disease. Acta Neuropathol. Commun. 2:12. 10.1186/2051-5960-2-12 24468113 PMC3912487

[B9] Chan J. P. Hu Z. Sieburth D. (2012). Recruitment of sphingosine kinase to presynaptic terminals by a conserved muscarinic signaling pathway promotes neurotransmitter release. Genes Dev. 26, 1070–1085. 10.1101/gad.188003.112 22588719 PMC3360562

[B10] Chan J. P. Sieburth D. (2012). Localized sphingolipid signaling at presynaptic terminals is regulated by calcium influx and promotes recruitment of priming factors. J. Neurosci. 32, 17909–17920. 10.1523/JNEUROSCI.2808-12.2012 23223309 PMC3545516

[B11] Chatzakos V. Rundlöf A. K. Ahmed D. de Verdier P. J. Flygare J. (2012). Inhibition of sphingosine kinase 1 enhances cytotoxicity, ceramide levels and ROS formation in liver cancer cells treated with selenite. Biochem. Pharmacol. 84, 712–721. 10.1016/j.bcp.2012.06.009 22727936

[B12] Chung N. Jenkins G. Hannun Y. A. Heitman J. Obeid L. M. (2000). Sphingolipids signal heat stress-induced ubiquitin-dependent proteolysis. J. Biol. Chem. 275, 17229–17232. 10.1074/jbc.C000229200 10764732

[B13] Cowart L. A. Gandy J. L. Tholanikunnel B. Hannun Y. A. (2010). Sphingolipids mediate formation of mRNA processing bodies during the heat-stress response of Saccharomyces cerevisiae. Biochem. J. 431, 31–38. 10.1042/BJ20100307 20629639 PMC3804835

[B14] Cui M. Wang Y. Cavaleri J. Kelson T. Teng Y. Han M. (2017). Starvation-induced stress response is critically impacted by ceramide levels in *Caenorhabditis elegans*. Genetics 205, 775–785. 10.1534/genetics.116.194282 27974500 PMC5289851

[B15] Cutler R. G. Thompson K. W. Camandola S. Mack K. T. Mattson M. P. (2014). Sphingolipid metabolism regulates development and lifespan in *Caenorhabditis elegans*. Mech. Ageing Dev. 143–144, 9–18. 10.1016/j.mad.2014.11.002 25437839 PMC4292899

[B16] Deng X. Yin X. Allan R. Lu D. D. Maurer C. W. Haimovitz-Friedman A.. (2008). Ceramide biogenesis is required for radiation-induced apoptosis in the germ line of *C. elegans*. Science 322, 110–115. 10.1126/science.1158111 18832646 PMC2585063

[B17] Di Pardo A. Amico E. Favellato M. Castrataro R. Fucile S. Squitieri F.. (2014). FTY720 (fingolimod) is a neuroprotective and disease-modifying agent in cellular and mouse models of Huntington disease. Hum. Mol. Genet. 23, 2251–2265. 10.1093/hmg/ddt615 24301680

[B18] Fayyaz S. Japtok L. Kleuser B. (2014). Divergent role of sphingosine 1-phosphate on insulin resistance. Cell. Physiol. Biochem. 34, 134–147. 10.1159/000362990 24977487

[B19] Feldman N. Kosolapov L. Ben-Zvi A. (2014). Fluorodeoxyuridine improves *Caenorhabditis elegans* proteostasis independent of reproduction onset. PLoS ONE 9:e85964. 10.1371/journal.pone.0085964 24465816 PMC3897603

[B20] Gangoiti P. Bernacchioni C. Donati C. Cencetti F. Ouro A. Gómez-Muñoz A.. (2012). Ceramide 1-phosphate stimulates proliferation of C2C12 myoblasts. Biochimie 94, 597–607. 10.1016/j.biochi.2011.09.009 21945811 PMC3314975

[B21] Guo L. Ou X. Li H. Han Z. (2014). Roles of sphingosine-1-phosphate in reproduction. Reprod. Sci. 21, 550–554. 10.1177/1933719113512534 24336672 PMC3984488

[B22] Hahm J.-H. Kim S. DiLoreto R. Shi C. Lee S.-J. V. Murphy C. T.. (2015). *C. elegans* maximum velocity correlates with healthspan and is maintained in worms with an insulin receptor mutation. Nat. Commun. 6, 8919. 10.1038/ncomms9919 26586186 PMC4656132

[B23] Hait N. C. Allegood J. Maceyka M. Strub G. M. Harikumar K. B. Singh S. K.. (2009). Regulation of histone acetylation in the nucleus by sphingosine-1-phosphate. Science 325, 1254–1257. 10.1126/science.1176709 19729656 PMC2850596

[B24] Hait N. C. Oskeritzian C. A. Paugh S. W. Milstien S. Spiegel S. (2006). Sphingosine kinases, sphingosine 1-phosphate, apoptosis and diseases. Biochim. Biophys. Acta Biomembr. 1758, 2016–2026. 10.1016/j.bbamem.2006.08.007 16996023

[B25] Hannun Y. A. Obeid L. M. (2008). Principles of bioactive lipid signalling: lessons from sphingolipids. Nat. Rev. Mol. Cell Biol. 9, 139–150. 10.1038/nrm2329 18216770

[B26] He X. Huang Y. Li B. Gong C. X. Schuchman E. H. (2010). Deregulation of sphingolipid metabolism in Alzheimer's disease. Neurobiol. Aging 31, 398–408. 10.1016/j.neurobiolaging.2008.05.010 18547682 PMC2829762

[B27] Herr D. R. Fyrst H. Creason M. B. Phan V. H. Saba J. D. Harris G. L. (2004). Characterization of the Drosophila sphingosine kinases and requirement for Sk2 in normal reproductive function. J. Biol. Chem. 279, 12685–12694. 10.1074/jbc.M310647200 14722126

[B28] Holland W. L. Brozinick J. T. Wang L. P. Hawkins E. D. Sargent K. M. Liu Y.. (2007). Inhibition of ceramide synthesis ameliorates glucocorticoid-, saturated-fat-, and obesity-induced insulin resistance. Cell Metab. 5, 167–179. 10.1016/j.cmet.2007.01.002 17339025

[B29] Holland W. L. Summers S. A. (2008). Sphingolipids, insulin resistance, and metabolic disease: new insights from *in vivo* manipulation of sphingolipid metabolism. Endocr. Rev. 29, 381–402. 10.1210/er.2007-0025 18451260 PMC2528849

[B30] Huang X. Withers B. R. Dickson R. C. (2014). Sphingolipids and lifespan regulation. Biochim. Biophys. Acta Mol. Cell Biol. Lipids 1841, 657–664. 10.1016/j.bbalip.2013.08.006 23954556 PMC3925463

[B31] Johnson K. R. Johnson K. Y. Becker K. P. Bielawski J. Mao C. Obeid L. M. (2003). Role of human sphingosine-1-phosphate phosphatase 1 in the regulation of intra- and extracellular sphingosine-1-phosphate levels and cell viability. J. Biol. Chem. 278, 34541–34547. 10.1074/jbc.M301741200 12815058

[B32] Kanno T. Nishizaki T. Proia R. L. Kajimoto T. Jahangeer S. Okada T.. (2010). Regulation of synaptic strength by sphingosine 1-phosphate in the hippocampus. Neuroscience 171, 973–980. 10.1016/j.neuroscience.2010.10.021 20950672

[B33] Kawabori M. Kacimi R. Karliner J. S. Yenari M. A (2013). Sphingolipids in cardiovascular and cerebrovascular systems: pathological implications and potential therapeutic targets. World J. Cardiol. 5, 75–86. 10.4330/wjc.v5.i4.75 23675553 PMC3653015

[B34] Keith S. A. Amrit F. R. G. Ratnappan R. Ghazi A. (2014). The, *C. elegans* healthspan and stress-resistance assay toolkit. Methods 68, 476–486. 10.1016/j.ymeth.2014.04.003 24727065

[B35] Kim Y. Sun H. (2012). ASM-3 Acid Sphingomyelinase functions as a positive regulator of the DAF-2/AGE-1 signaling pathway and serves as a novel anti-aging target. PLoS ONE 7:e45890. 10.1371/journal.pone.0045890 23049887 PMC3457945

[B36] Kitatani K. Idkowiak-Baldys J. Bielawski J. Taha T. A. Jenkins R. W. Senkal C. E.. (2006). Protein kinase C-induced activation of a ceramide/protein phosphatase 1 pathway leading to dephosphorylation of p38 MAPK. J. Biol. Chem. 281, 36793–36802. 10.1074/jbc.M608137200 17030510

[B37] Kitatani K. Idkowiak-Baldys J. Hannun Y. A. (2008). The sphingolipid salvage pathway in ceramide metabolism and signaling. Cell. Signal. 20, 1010–1018. 10.1016/j.cellsig.2007.12.006 18191382 PMC2422835

[B38] Levkau B. (2013). Cardiovascular effects of sphingosine-1-phosphate (S1P). Handb. Exp. Pharmacol. 216, 147–170. 10.1007/978-3-7091-1511-4_8 23563656

[B39] Liu J. Huang X. Withers B. R. Blalock E. Liu K. Dickson R. C. (2013). Reducing sphingolipid synthesis orchestrates global changes to extend yeast lifespan. Aging Cell 12, 833–841. 10.1111/acel.12107 23725375 PMC3773046

[B40] Liu Y. Samuel B. S. Breen P. C. Ruvkun G. (2014). *Caenorhabditis elegans* pathways that surveil and defend mitochondria. Nature 508, 406–410. 10.1038/nature13204 24695221 PMC4102179

[B41] Maceyka M. Harikumar K. B. Milstien S. Spiegel S. (2012). Sphingosine-1-phosphate signaling and its role in disease. Trends Cell Biol. 22, 50–60. 10.1016/j.tcb.2011.09.003 22001186 PMC3253987

[B42] Maceyka M. Milstien S. Spiegel S. (2007). Shooting the messenger: oxidative stress regulates sphingosine-1-phosphate. Circ. Res. 100, 7–9. 10.1161/01.RES.0000255895.19868.a3 17204658

[B43] Mendel J. Heinecke K. Fyrst H. Saba J. D. (2003). Sphingosine phosphate lyase expression is essential for normal development in *Caenorhabditis elegans*. J. Biol. Chem. 278, 22341–22349. 10.1074/jbc.M302857200 12682045

[B44] Menuz V. Howell K. S. Gentina S. Epstein S. Riezman I. Fornallaz-Mulhauser M.. (2009). Protection of *C. elegans* from anoxia by HYL-2 ceramide synthase. Science 324, 381–384. 10.1126/science.1168532 19372430

[B45] Mitsutake S. Yokose U. Kato M. Matsuoka I. Yoo J.-M. Kim T.-J.. (2007). The generation and behavioral analysis of ceramide kinase-null mice, indicating a function in cerebellar Purkinje cells. Biochem. Biophys. Res. Commun. 363, 519–524. 10.1016/j.bbrc.2007.09.010 17888878

[B46] Morita Y. Perez G. I. Paris F. Miranda S. R. Ehleiter D. Haimovitz-Friedman A. (2000). Oocyte apoptosis is suppressed by disruption of the acid sphingomyelinase gene or by sphingosine-1-phosphate therapy. Nat. Med. 6, 1109–1114. 10.1038/80442 11017141

[B47] Mosbech M. B. Kruse R. Harvald E. B. Olsen A. S. B. Gallego S. F. Hannibal-Bach H. K.. (2013). Functional loss of two ceramide synthases elicits autophagy-dependent lifespan extension in *C. elegans*. PLoS ONE 8:e70087. 10.1371/journal.pone.0070087 23894595 PMC3716707

[B48] Muir J. L. (1997). Acetylcholine, aging, and Alzheimer's disease. Pharmacol. Biochem. Behav. 56, 687–696. 10.1016/S0091-3057(96)00431-5 9130295

[B49] Mulcahy B. Holden-Dye L. O'Connor V. (2012). Pharmacological assays reveal age-related changes in synaptic transmission at the *Caenorhabditis elegans* neuromuscular junction that are modified by reduced insulin signalling. J. Exp. Biol. 216(Pt 3), 492–501. 10.1242/jeb.068734 23038730

[B50] Okada T. Kajimoto T. Jahangeer S. Nakamura S. (2009). Sphingosine kinase/sphingosine 1-phosphate signalling in central nervous system. Cell. Signal. 21, 7–13. 10.1016/j.cellsig.2008.07.011 18694820

[B51] Olivera A. Rosenfeldt H. M. Bektas M. Wang F. Ishii I. Chun J.. (2003). Sphingosine kinase type 1 induces G12/13-mediated stress fiber formation, yet promotes growth and survival independent of G protein-coupled receptors. J. Biol. Chem. 278, 46452–46460. 10.1074/jbc.M308749200 12963721

[B52] Panneer Selvam S. De Palma R. M. Oaks J. J. Oleinik N. Peterson Y. K. Stahelin R. V.. (2015). Binding of the sphingolipid S1P to hTERT stabilizes telomerase at the nuclear periphery by allosterically mimicking protein phosphorylation. Sci. Signal. 8:ra58. 10.1126/scisignal.aaa4998 26082434 PMC4492107

[B53] Pchejetski D. Kunduzova O. Dayon A. Calise D. Seguelas M. H. Leducq N.. (2007). Oxidative stress-dependent sphingosine kinase-1 inhibition mediates monoamine oxidase a-associated cardiac cell apoptosis. Circ. Res. 100, 41–49. 10.1161/01.RES.0000253900.66640.34 17158340

[B54] Pitson S. M. (2011). Regulation of sphingosine kinase and sphingolipid signaling. Trends Biochem. Sci. 36, 97–107. 10.1016/j.tibs.2010.08.001 20870412

[B55] Pitson S. M. Moretti P. A. B. Zebol J. R. Xia P. Gamble J. R. Vadas M. A.. (2000). Expression of a catalytically inactive sphingosine kinase mutant blocks agonist-induced sphingosine kinase activation. a dominant-negative sphingosine kinase. J. Biol. Chem. 275, 33945–33950. 10.1074/jbc.M006176200 10944534

[B56] Pyszko J. Strosznajder J. B. (2014). Sphingosine kinase 1 and sphingosine-1-phosphate in oxidative stress evoked by 1-methyl-4-phenylpyridinium (MPP+) in human dopaminergic neuronal cells. Mol. Neurobiol. 50, 38–48. 10.1007/s12035-013-8622-4 24399507

[B57] Romero-Guevara R. Cencetti F. Donati C. Bruni P. (2015). Sphingosine 1-phosphate signaling pathway in inner ear biology. new therapeutic strategies for hearing loss? Front. Aging Neurosci. 7:60. 10.3389/fnagi.2015.00060 25954197 PMC4407579

[B58] Samuelson A. V. Carr C. E. Ruvkun G. (2007). Gene activities that mediate increased life span of *C. elegans* insulin-like signaling mutants. Genes Dev. 21, 2976–2994. 10.1101/gad.1588907 18006689 PMC2049198

[B59] Shen H. Giordano F. Wu Y. Chan J. Zhu C. Milosevic I.. (2014). Coupling between endocytosis and sphingosine kinase 1 recruitment. Nat. Cell Biol. 16, 652–662. 10.1038/ncb2987 24929359 PMC4230894

[B60] So S. Tokumaru T. Miyahara K. Ohshima Y. (2011). Control of lifespan by food bacteria, nutrient limitation and pathogenicity of food in *C. elegans*. Mech. Ageing Dev. 132, 210–212. 10.1016/j.mad.2011.02.005 21354440

[B61] Sowa J. N. Mutlu A. S. Xia F. Wang M. C. (2015). Olfaction modulates reproductive plasticity through neuroendocrine signaling in *Caenorhabditis elegans*. Curr. Biol. 25, 2284–2289. 10.1016/j.cub.2015.07.023 26279229 PMC4825799

[B62] Spiegel S. Milstien S. (2003). Sphingosine-1-phosphate: an enigmatic signalling lipid. Nat. Rev. Mol. Cell Biol. 4, 397–407. 10.1038/nrm1103 12728273

[B63] Tani K. Tabuchi K. Hara A. (2016). Hair cell loss induced by sphingosine and a sphingosine kinase inhibitor in the Rat Cochlea. Neurotox. Res. 29, 35–46. 10.1007/s12640-015-9563-7 26472207

[B64] Taniguchi M. Kitatani K. Kondo T. Hashimoto-Nishimura M. Asano S. Hayashi A.. (2012). Regulation of autophagy and its associated cell death by “sphingolipid rheostat”: reciprocal role of ceramide and sphingosine 1-phosphate in the mammalian target of rapamycin pathway. J. Biol. Chem. 287, 39898–39910. 10.1074/jbc.M112.416552 23035115 PMC3501064

[B65] Tedesco P. Jiang J. Wang J. Jazwinski S. M. Johnson T. E. (2008). Genetic analysis of hyl-1, the *C. elegans* homolog of LAG1/LASS1. Age 30, 43–52. 10.1007/s11357-008-9046-3 19424872 PMC2274941

[B66] Van Brocklyn J. R. Williams J. B. (2012). The control of the balance between ceramide and sphingosine-1-phosphate by sphingosine kinase: oxidative stress and the seesaw of cell survival and death. Comp. Biochem. Physiol. B Biochem. Mol. Biol. 163, 26–36. 10.1016/j.cbpb.2012.05.006 22613819

[B67] Van Echten-Deckert G. Walter J. (2012). Sphingolipids: critical players in Alzheimer's disease. Prog. Lipid Res. 51, 378–393. 10.1016/j.plipres.2012.07.001 22835784

[B68] Vecer J. Vesela P. Malinsky J. Herman P. (2014). Sphingolipid levels crucially modulate lateral microdomain organization of plasma membrane in living yeast. FEBS Lett. 588, 443–449. 10.1016/j.febslet.2013.11.038 24333335

[B69] Wu D. Ren Z. Pae M. Guo W. Cui X. Merrill A. H.. (2007). Aging up-regulates expression of inflammatory mediators in mouse adipose tissue. J. Immunol. 179, 4829–4839. 10.4049/jimmunol.179.7.4829 17878382

[B70] Yang J.-S. S. Nam H. G. H.-J. J. Seo M. Han S. K. Choi Y. Nam H. G. H.-J. J.. (2011). OASIS: online application for the survival analysis of lifespan assays performed in aging research. PLoS ONE 6:e23525. 10.1371/journal.pone.0023525 21858155 PMC3156233

[B71] Youm J. K. Jo H. Hong J. H. Shin D. M. Kwon M. J. Jeong S. K.. (2008). K6PC-5, a sphingosine kinase activator, induces anti-aging effects in intrinsically aged skin through intracellular Ca2+ signaling. J. Dermatol. Sci. 51, 89–102. 10.1016/j.jdermsci.2008.03.002 18420384

